# TTK/hMPS1 Is an Attractive Therapeutic Target for Triple-Negative Breast Cancer

**DOI:** 10.1371/journal.pone.0063712

**Published:** 2013-05-20

**Authors:** Virginie Maire, Céline Baldeyron, Marion Richardson, Bruno Tesson, Anne Vincent-Salomon, Eléonore Gravier, Bérengère Marty-Prouvost, Leanne De Koning, Guillem Rigaill, Aurélie Dumont, David Gentien, Emmanuel Barillot, Sergio Roman-Roman, Stéphane Depil, Francisco Cruzalegui, Alain Pierré, Gordon C. Tucker, Thierry Dubois

**Affiliations:** 1 Institut Curie, Research Center, Paris, France; 2 Breast Cancer Biology Group, Department of Translational Research, Paris, France; 3 Tumor Biology, Service of Pathology, Paris, France; 4 INSERM U900, Bioinformatics, Biostatistics, Epidemiology and Computational Systems Biology of Cancer, Paris, France; 5 Mines ParisTech, Fontainebleau, France; 6 RPPA platform, Department of Translational Research, Paris, France; 7 AgroParisTech/INRA, UMR 518, MIA, Paris, France; 8 Platform of Molecular Biology Facilities, Department of Translational Research, Paris, France; 9 Oncology Research and Development Unit, Institut de Recherches SERVIER, Croissy-sur-Seine, France; Sun Yat-sen University Medical School, China

## Abstract

Triple-negative breast cancer (TNBC) represents a subgroup of breast cancers (BC) associated with the most aggressive clinical behavior. No targeted therapy is currently available for the treatment of patients with TNBC. In order to discover potential therapeutic targets, we searched for protein kinases that are overexpressed in human TNBC biopsies and whose silencing in TNBC cell lines causes cell death. A cohort including human BC biopsies obtained at Institut Curie as well as normal tissues has been analyzed at a gene-expression level. The data revealed that the human protein kinase monopolar spindle 1 (hMPS1), also known as TTK and involved in mitotic checkpoint, is specifically overexpressed in TNBC, compared to the other BC subgroups and healthy tissues. We confirmed by immunohistochemistry and reverse phase protein array that TNBC expressed higher levels of TTK protein compared to the other BC subgroups. We then determined the biological effects of TTK depletion by RNA interference, through analyses of tumorigenic capacity and cell viability in different human TNBC cell lines. We found that RNAi-mediated depletion of TTK in various TNBC cell lines severely compromised their viability and their ability to form colonies in an anchorage-independent manner. Moreover, we observed that TTK silencing led to an increase in H2AX phosphorylation, activation of caspases 3/7, sub-G1 cell population accumulation and high annexin V staining, as well as to a decrease in G1 phase cell population and an increased aneuploidy. Altogether, these data indicate that TTK depletion in TNBC cells induces apoptosis. These results point out TTK as a protein kinase overexpressed in TNBC that may represent an attractive therapeutic target specifically for this poor prognosis associated subgroup of breast cancer.

## Introduction

Breast cancer (BC) is one of the most common human malignancies, accounting for 22% of all cancers diagnosed in women. BC represents a complex and heterogeneous disease comprising distinct pathologies with specific histological features, therapeutic responses, metastatic dissemination patterns and patient outcomes. During the last decade, global gene-expression analyses have revealed four main distinct subgroups in human breast tumors: luminal A and luminal B (LA and LB), human epidermal growth factor receptor 2-overexpressing (Her2) and basal-like (BLBC) breast cancers [1]–[4]. LA and LB express hormone receptor-related genes, estrogen receptor (ER) and progesterone receptor (PR). BLBC (and triple-negative breast cancers (TNBC)) are characterized by the absence of ER and PR expression and the lack of Her2 overexpression [5]. In addition, BLBC are positive for the expression of basal cytokeratins (CK5/6, CK14) and/or epidermal growth factor receptor (EGFR) [6]–[8].

Of all the BC subgroups, TNBC represent the greatest clinical challenge because these tumors are prevalent in younger women, associated with the worst prognosis and often relapse rapidly [9]. TNBC are highly proliferative, genetically unstable, poorly differentiated, often grade III carcinomas [9], [10] and preferentially metastasize to brain and lung [11]. In contrast to ER-positive luminal tumors and Her2 carcinomas, which can be treated with targeted therapies such as tamoxifen (estrogen antagonist), aromatase inhibitors or anti-Her2 monoclonal antibodies [12], [13], there is no available targeted therapy for TNBC. Patients with TNBC are treated exclusively with conventional cytotoxic therapies. While they show high rates of objective initial response, the majority of patients do not have a complete and prolonged response and are at high risk for relapse and death within the first 3–5 years of diagnosis [10], [14], [15]. Whereas some molecules are in clinical trials in patients with TNBC, such as dasatinib (Src inhibitor), cetuximab (EGFR inhibitor), bevacizumab (vascular endothelial growth factor inhibitor) or olaparib (Poly[ADP-ribose] polymerase inhibitor) [16], identification of relevant molecular targets in TNBC remains a critical challenge.

In order to discover potential therapeutic targets, we searched for protein kinases that are overexpressed in human TNBC biopsies, and which cause cell death when inhibited in TNBC cell lines. From gene expression analyses performed on a cohort of normal breast tissues and human BC biopsies obtained from Institut Curie, where all BC subtypes are almost equally represented, we found that RNA transcript levels of the human protein kinase monopolar spindle 1 (hMps1/TTK) gene, which encodes a dual serine/threonine kinase involved in the mitotic spindle assembly checkpoint (SAC) [17], [18], are highly increased in TNBC samples compared to the other BC subgroups and normal tissue samples. High levels of TTK mRNA have been found in BC [19], particularly in TNBC [20], [21] where it has been shown to protect cancer cells from aneuploidy [20]. The SAC ensures the faithful segregation of sister chromatids over the two daughter cells by inhibiting progression from metaphase to anaphase, until all sister chromatids are attached to microtubules of the mitotic spindle in a bipolar orientation [22]. The absence of a functional SAC results in gross chromosome mis-segregation leading to cell death [23], [24], suggesting that inhibition of this mitotic checkpoint could have therapeutic potential in cancer treatment [25], [26].

We focused on the potential deleterious effect of TTK depletion on TNBC cell viability. To address this issue, we demonstrated first that TTK is overexpressed in TNBC compared to the other BC subgroups at the protein level by immunohistochemistry (IHC) and reverse phase protein array (RPPA) techniques. We found that the RNAi-mediated depletion of TTK severely compromises viability of human TNBC cells and their capacity to form colonies in an anchorage-independent manner. Moreover, TTK depletion in TNBC cell lines leads to an increase in H2AX phosphorylation, an accumulation of cells in sub-G1 phase and cell death by apoptosis. Together, these results highlight TTK as a potential therapeutic target for the TNBC subgroup of breast cancer.

## Methods

### Cell Culture

We obtained all cell lines used in this study from the American Type Culture Collection (ATCC, LGC Promochem), except MDA-MB-231 cells provided by Mina Bissell (Life Sciences Division, Lawrence Berkeley National Laboratory, Berkeley, California, USA). We maintained BT-549, HCC1428, HCC1569, HCC1954, HCC202, MDA-MB-468 and ZR-75-1 cells (HTB-122, CRL-2327, CRL-2330, CRL-2338, CRL-2316, HTB-132 and CRL-1500) in RPMI-1640 (Invitrogen) supplemented with 10% (vol/vol) fetal bovine serum (FBS, Invitrogen), 100 U/ml penicillin and 100 µg/ml streptomycin (P/S, Invitrogen). We used the same medium complemented (i) with 1.5 g/l sodium bicarbonate, 10 mM Hepes (Invitrogen) and 1 mM sodium pyruvate (Invitrogen) for HCC1143, HCC1187, HCC1937, HCC38 and HCC70, cells (CRL-2321, CRL-2322, CRL-2336, CRL-2314 and CRL-2315) or (ii) with 10% FBS and 0.2 U/ml bovine insulin (Sigma-Aldrich) for T47D cells (HTB-133). We cultured MDA-MB-231 cells in DMEM-F12 (Invitrogen) supplemented with 10% FBS and P/S. 184B5 and MCF10A cells (CRL-8799 and CRL-10317) were grown in the same medium supplemented with 5% (vol/vol) horse serum (HS; Invitrogen), 20 ng/ml EGF (Sigma-Aldrich), 100 ng/ml cholera toxin (Sigma-Aldrich), 0.01 mg/ml insulin, 500 ng/ml hydrocortison (SERB Laboratories). We used the same mix of medium in which we added 1.2 g/l sodium bicarbonate, 0.5 mM sodium pyruvate and 15 mM Hepes for MCF12A cells (CRL-10782). Hs 578T, MDA-MB-134-VI and MDA-MB-361 cells (HTB-126, HTB-23 and HTB-27) were maintained in DMEM (Invitrogen) supplemented with 10% FBS and P/S. We grew BT-20 cells (HTB-19) in MEM (Sigma-Aldrich) containing 10% FBS, 1.5 g/l sodium bicarbonate (Invitrogen), 0.1 mM non-essential amino-acids (NEAA, Invitrogen) and 1 mM sodium pyruvate (Invitrogen). We used Leibovitz's L-15 medium (Invitrogen) containing P/S and 10 mM Hepes, supplemented with 10% FBS for MDA-MB-157, MDA-MB-436 and MDA-MB-453 cells (HTB-24, HTB-130 and HTB-131). We cultured SK-BR-3 cells (HTB-30) in McCoy5a (Invitrogen) containing 10% FBS and P/S. We maintained all cell lines at 37°C in a humidified atmosphere with 5% CO_2_.

### Tissue Collection and Immunohistochemistry

We obtained 154 tumor samples (DNA, RNA and biopsies) from patients treated at the hospital of Institut Curie (Biological Resource Center, Paris, France). In addition, 18 healthy samples were obtained from mammary plastic surgery. Experiments were performed in agreement with the Bioethic Law No. 2004-800 and the Ethic Charter from the French National Institute of Cancer (INCa), and after approval of the ethics committee of our Institution. Informed consents were not required, women were informed of the research use of their tissues, and did not declare any opposition for such researches. We performed IHC on these tissues, as described previously [27] and we determined after haematoxylin-eosin-safran (HES) staining that the tumors contained between 50–90% cancerous cells. We recorded ER and PR positive nuclear staining in accordance with standardized guidelines, using 10% as the cut-off for ER and PR positive cells. For Her2-positivity, we considered only staining of membranes with a 30% cut-off as recommended in [28]. From IHC data, our cohort consists of 35 LA (ER+, PR+, Her2–); 40 LB (ER+, PR+, Her2+/−); 33 Her2 (ER–, PR–, Her2+) and 46 TNBC (ER–, PR-, Her2–). Their grades were as follows: 34 grade I and 1 grade II for LA; 2 grade II and 38 grade III for LB; 1 grade II and 32 grade III for Her2 and 46 grade III for TNBC.

For TTK staining, we built tissue microarrays (TMA) containing alcohol, formalin and acetic acid (AFA)-fixed paraffin-embedded tissue as described [29]. For each biopsy, we carefully selected three representative tumor areas and one peri-tumoral tissue from a HES-stained section of a donor block. Using a specific arraying device (Manual Tissue Arrayer, Beecher Instruments), we punched core cylinders of 1 mm in diameter from each of those four areas and placed them into recipient paraffin blocks. We cut sections of 3 µm, placed them onto positively charged slides (capillary gap microscope slides, Dako) and dried them at 58°C for one hour. We deparaffinized the sections in toluene and hydrated them in graded alcohol. Then, we performed the antigen retrieval step in 10 mM sodium citrate pH 6.10 or 10 mM EDTA pH 8 (LabVision, Thermo Scientific) for 20 min at 95°C. We blocked endogenous biotins by Biotin blocking system (Dako). After washes in PBS containing 0.05% Tween 20, we quenched endogenous peroxidase activity with 3% hydrogen peroxide for 5 min and rinsed in distilled water. We blocked each tissue section with PBS containing 1% bovin serum albumin (BSA, Sigma) and 1.4% normal donkey serum (Jackson ImmunoResearch Laboratories, Inc., Interchim) for 5 min and incubated them overnight at 4°C with the appropriate primary antibody. After washes, we incubated slides with the appropriate biotinylated secondary antibody for 30 min at room temperature (RT). We revealed immunostaining by using the Vectastain ABC peroxidase system (Vector Laboratories; Abcys), using diaminobenzidine (DAB) as a chromogen. Moreover, we counter-stained slides with haematoxylin before mounting. We carried out all reactions using an automated stainer (LabVision; Thermo Scientific), except for the primary antibody. Negative controls consisted in the omission of the primary antibody. We first optimized IHC conditions using cell pellets from cell lines expressing high or low levels of TTK.

### Clinical Studies

We defined as disease-free interval (DFI) the time from the diagnosis of BC to the occurrence of a locoregional, distant or contralateral recurrence. Patients with initial metastatic disease or with a previous aggressive cancer were excluded; and 39 TNBC, 28 Her2, 28 LA and 25 LB were thus analyzed. For the analysis specifically within the TNBC subgroup, we assessed the correlation between TTK expression and prognosis by including TTK expression as a continuous variable (no bimodal distribution of TTK expression was observed within the TNBC subgroup) in a Cox proportional-hazards regression model, and significance was assessed using Wald test. In order to illustrate the correlation reported by the Wald test, Kaplan–Meier survival plots and logrank tests were used to compare prognosis for tumors with high and low TTK gene expression (dichotomized at the median).

### DNA Microarray Analysis

We analyzed TTK DNA copy number in the different tumor subtypes (46 TNBC; 33 Her2; 39 LB and 35 LA) and in 17 healthy tissue samples. We purified DNA from frozen tumor samples with the use of a standard phenol/chloroform procedure, as described in [30] and hybridized 500 ng of genomic DNA onto Affymetrix *SNP6.0* microarrays, according to the manufacturer recommendations. We first normalized and analyzed data from *SNP6.0* microarrays using Affymetrix Genotyping Console. Next we performed a segmentation of the signal from SNP probes using the Colibri function available in the CGHseg R package [31] to identify copy number alterations. In a first segmentation round, we identified and discarded outlier probes giving rise to singleton segments. After a second segmentation round, we merged segments that contained less than five probes to the closest adjacent segment. For every sample, we produced a smoothed signal by setting the values of all probes within one segment to the average of all probes on that segment. We defined the thresholds for gain and loss as the.999 and.001 quantiles of the distribution of smoothed probe signal levels from the healthy breast tissue samples.

### RNA Microarray Analysis

We analyzed the expression of TTK at the mRNA level in the different tumor subtypes (41 TNBC; 30 Her2; 30 LB and 29 LA) and in 11 healthy tissue samples. We extracted and purified total RNA from frozen tumor samples by using the RNeasy Mini Kit (Qiagen) and then, the RNA clean up kit (Macherey Nagel), according to the manufacturers’ instructions. After RNA quality and quantity controls, 2 µg of total RNA were amplified and labeled according to the Affymetrix one cycle synthesis target preparation protocol to hybridize Affymetrix U133 plus 2.0 chips, as described by the company. We analyzed data by using the brainarray HGU133Plus2_Hs_ENTREZG version 13 custom chipset definition file [32]. We first normalized transcriptomic data by using GC-RMA (version 2.14.1) [33]. We then fitted a linear mixed model by using the nlme package using Restricted Maximum Likelihood (REML) [34]. The model included the different experimental batches and the sample type as fixed effects, and treated technical variation as random effects. No interaction terms were included. We used this model to derive significance for differential expression between different tumor types and for correction of batch and hybridation effects. For further analysis, we averaged technical replicates. From the distribution of the GC-RMA normalized data we observed a clear bi-modal distribution of intensities, which can be interpreted as the presence of a large number of probes with noise-level signal. We chose to discard such probe-sets from this analysis: specifically, we considered as not expressed and therefore discarded, all the probe-sets with a log2 intensity of less than 4 in 95% or more of tissue samples. This filter reduced the number of probe-sets from 18123 to 11543, each uniquely matching an Entrez gene entry.

### Protein Extracts

We lysed the cells or the frozen tumors in Laemmli buffer containing 50 mM Tris pH 6.8, 2% SDS, 5% glycerol, 2 mM DTT, 2.5 mM EDTA, 2.5 mM EGTA, 2 mM sodium orthovanadate and 10 mM sodium fluoride (Sigma-Aldrich), supplemented with a cocktail of protease (Roche) and phosphatase (Thermo Scientific) inhibitors, as described previously [29]. Specifically, we obtained homogenization of tumors by using a TissueLyser (Qiagen) with stainless steel beads 5 mm in diameter (Qiagen) for 2–3 min at 30 Hz. Subsequently, we boiled cell or tumor lysates at 100°C for 10 minutes. We quantified the protein concentration by using the BCA Protein Assay Kit-Reducing Agent Compatible (Pierce, Perbio).

### Reverse Phase Protein Array

We analyzed the expression of TTK at the protein level in the different tumor subtypes (42 TNBC; 29 Her2; 37 LB and 24 LA) by Reverse phase protein array (RPPA) technology, as described previously [29], with some modifications. We deposited protein samples in five 2-fold serial dilutions starting from at least 2 ng of protein lysates per spot onto nitrocellulose covered slides (Schott Nexterion NC-C) using a dedicated arrayer (Aushon Biosystems 2470). After printing, we incubated slides, using the Autostainer Plus automaton (Dako), with avidin, biotin and peroxydase blocking reagents (Dako) before saturation with TBS containing 0.1% Tween 20 (TBST) and 5% BSA (TBST-BSA). We then probed slides overnight at 4°C with the appropriate primary antibodies (or without primary antibody for negative control) diluted in TBST-BSA. After washes with TBST, we probed the arrays with the appropriate horseradish peroxidase-coupled (HRP) secondary antibodies diluted in TBST-BSA for one hour at RT. To amplify the signal, we incubated the slides with Bio-Rad Amplification Reagent (BAR solution, BioRad) for 15 minutes at RT and washed them with TBST. To detect the bound biotin, we probed the arrays with alexa 647-conjugated streptavidin (1∶1,000; S-32357, Molecular Probe) diluted in TBST-BSA for one hour at RT and washed them in TBST.

We dried the processed slides by centrifugation and scanned them using a GenePix 4000B microarray scanner (Molecular Devices). We determined spot intensity with MicroVigene software (VigeneTech Inc). The data obtained for TTK was part of a larger set of 27 RPPA arrays that we used to analyze the same tumor samples and cell lines with different antibodies. Briefly, the data from each RPPA slide was log2 transformed, median centered and scaled (divided by median absolute deviation). We then adjusted sample loadings effects individually for each hybridization by correcting the dependency of the data for individual arrays on the median value of each sample over all 27 arrays using a linear regression. These preprocessing steps (centering, scaling and sample loading correction) were done separately for tumor samples and for cell lines. We used T tests to assess the significance of the differential abundance in TTK between different BC subgroups.

### Immunoblot (IB)

For each extract, we separated 10 µg of protein on 4–12% TGX gels (BioRad) and transferred onto nitrocellulose membranes (BioRad). We saturated membranes with TBST-BSA and incubated them overnight at 4°C with the appropriate primary antibodies diluted in TBST-BSA. After washes, we incubated membranes with the appropriate HRP peroxidase secondary antibodies for one hour at room temperature. For visualization of proteins, we incubated membranes with ECL solution (Amersham, GE Healthcare) for chemiluminescence detection and imaged membranes using the LAS-3000 Luminescent Image analyser and Image Gauge software (Fuji, FSVT).

### Antibodies

Primary antibodies used during IHC, IB and RPPA experiments are listed below:

Rabbit anti-β-actin (IB, 1/5,000; A2668, Sigma-Aldrich) [29], which was used as loading control; rabbit anti-cleaved caspase 7 (IB, 1∶1,000; 9491, Cell Signaling Technology) [29]; mouse anti-CK5/6 (IHC, 1∶50; clone D5/16B4, Dako) [29]; mouse anti-CK14 (IHC, prediluted; clone LL002, Biogenex) [29]; mouse anti-EGFR (IHC, 1∶40; 08-1205, clone 31G7, Zymed) [29]; ER (IHC, 1∶50; clone 6F11/2, Novocastra) [35]; mouse anti-γH2AX (IB- 1∶2,000; 05-636; Millipore) [36]; Her2 (IHC, 1∶800; clone CB11, Novocastra) [37]; mouse anti-cleaved PARP (IB, 1∶1,000; 9546, Cell Signaling Technology) [29]; PR (IHC, 1∶200; clone 1A6, Novocastra) [35]; mouse anti-TTK (IHC, 1∶25; RPPA, 1∶250 and IB, 1∶500; sc-56968, Santa Cruz) [38].

Secondary antibodies used were: For IHC, biotinylated donkey anti-mouse or anti-rabbit (1∶500; Jackson ImmunoResearch Laboratories, Inc., Interchim); for IB or RPPA, horseradish peroxidase-conjugated affinity-purified goat anti–mouse or anti–rabbit (1∶20,000; Jackson ImmunoResearch Laboratories, Inc., Interchim).

### siRNA and Transfection

According to the subsequent experiments, we seeded cells into 6- or 96-well plates at a density determined on the basis of the growth characteristics of each cell line. We thus plated respectively 75,000 and 1,000 cells for MCF10A; 150,000 and 1,500 cells for MDA-MB-231; 200,000 and 3,000 cells for MDA-MB-468 and 300,000 and 8,000 cells for HCC70. With the exception of HCC70 cells, which were plated and transfected at the same time, we transfected cells a day after plating with 20 nM siRNA duplexes, either a control siRNA (siCtrl; 1027281, Qiagen) or 2 distinct siRNAs against TTK, TTK_6, 5′-CAGCAATACCTTGGATGATTA-3′ and TTK_7, 5′-TCCGACTTTATGATTATGAAA-3′ (SI02223207 and SI02223214, Qiagen) using Lipofectamine RNAiMAX reagent (Invitrogen) in Opti-MEM medium (Invitrogen), according to the manufacturers’ instructions. We harvested cells at various time points (see legend of figures).

### Cell Proliferation Assay

We determined the ability of cells to grow using a colorimetric assay based on the reduction of 3-(4,5-dimethylthiazol-2-yl)-2,5 diphenyltetrazolium bromide (MTT; M-2128, Sigma) to formazan within the mitochondria of living cells. To the medium in each well, we added 15 µl of MTT (5 mg/ml dissolved in PBS). After 4 h incubation at 37°C, we lysed floating plus adherent cells with 100 µl of 10% SDS in 10 mM HCl. We measured absorbance at the wavelength of 540 nm on an Infinite 200 spectrophotometer (Tecan). We performed all experiments in quadruplicate and presented the results as a percentage of cell proliferation normalized to control siRNA condition, as in [29].

### Cell Cycle Analysis by Flow Cytometry

We collected siRNA-treated floating and detached (after trypsinization) cells. Then, we washed them once with PBS and then with PBS containing 0.5% BSA. We fixed the cells in cold 70% ethanol with gentle vortexing. After fixation, we incubated the cells in PBS containing 10 µg/ml propidium iodide (PI; P3566, Invitrogen) and 200 µg/ml RNase A (Pure Link™ RNase A, Invitrogen) for 30 min at RT. We collected the samples using FACScalibur (Becton Dickinson) and we analyzed a minimum of 20,000 cells per sample using CellQuest software (Becton Dickinson). We quantified DNA content by using Modfit LT software (Verity Software House) and we expressed results as a distribution of cells in each cell-cycle phase plus sub-G1 population.

### Apoptosis Evaluation

Following siRNA-mediated depletion, we harvested cells at the indicated time points (see legend of figures) and we detected apoptosis using the following assays:

Caspase 3/7 activation: We evaluated caspase activity by using Caspase-Glo® 3/7 luminescent assay (Promega), according to the manufacturers’ instructions, as in [29]. We performed experiments in triplicate and presented results as caspase 3/7 activity normalized by caspase 3/7 activity from siCtrl-transfected cells.

Detection of PARP and caspase 7 cleavages: We performed immunoblot (see corresponding paragraph above) using whole protein lysates of floating plus adherent cells to visualize the cleavages of PARP and capase 7, which serve as markers of cells undergoing apoptosis.

Annexin V assay: Additionally, we determined the proportion of apoptotic cells by using the annexin-V-FLUOS staining kit (Roche) according to the manufacturer’s instructions. After sequential staining by annexin V and PI, we performed flow cytometry analyses on a LSRII Instrument (Becton Dickinson). Using CellQuest software, we analyzed a minimum of 10,000 cells per sample and we evaluated the percentage of living cells with low annexin V and low PI staining, apoptotic cells with high annexin V and low PI staining and necrotic cells with high annexin V and high PI staining.

### Soft-agar Tumorigenicity Assay

A day after siRNA transfection, we trypsinized MDA-MB-231, MDA-MB-468 and HCC70 cells. We resuspended 5×10^3^ cells in 0.35% soft-agar medium, consisting of equal volumes of 0.70% agarose (A4018, Sigma) and 2X culture medium, and plated onto 1 ml of solidified 0.5% soft-agar in 6-well plates. For MDA-MB-231 cells, which are extremely invasive, we added, on top of the cells, an additional layer of 0.5% agar medium. We incubated all cell lines for 4 weeks at 37°C in a humidified atmosphere with 5% CO_2_. We stained cell colonies by MTT. We automatically quantified the number of colonies by using the LAS-3000 Luminescent Image analyser (Fuji, FSVT) and ImageJ 1.43u software (NIH). We performed all experiments in triplicate and presented the results as a percentage of colonies normalized to colonies from siCtrl-transfected cells.

## Results

### High Expression of TTK is Associated with Triple-negative Breast Cancers

We have constituted our tumor collection to get a similar number of biopsies for each of the 4 main breast cancer subgroups. Biopsies (46 TNBC, 33 Her2, 35 LA and 40 LB), obtained from patients treated at the hospital of Institut Curie (Biological Resource Center), were characterized by IHC (data not shown). We confirmed by microarray-based transcriptional profiling analyses that the breast cancer subtypes identified by IHC also clustered according to BLBC/TNBC, Her2, LA and LB breast cancer gene-expression signatures [39] (data not shown). In addition, our BC cohort, biased to contain all subtypes equally represented and accurately characterized (see materials and methods), contains 18 healthy breast samples obtained from reduction mammoplasty.

**Figure 1:**
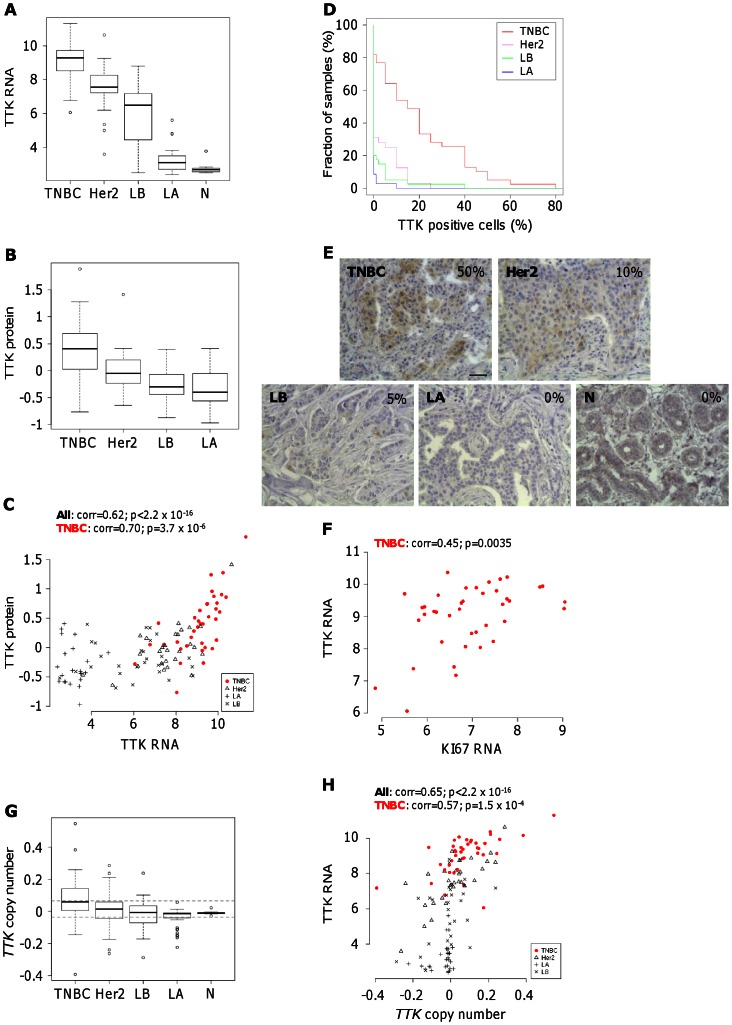
High expression of TTK in human triple-negative breast cancers. (**A and B**) *RNA and protein levels of TTK in human breast cancers from omic data selected from the Institut Curie Human Tumor database.* Box-plots illustrate the logarithmic (log2) transformed levels of TTK RNA (A) and protein (B) expression in triple-negative breast cancer (TNBC), Her2-overexpressing/ER-negative tumors (Her2), luminal A (LA) and luminal B (LB) tumors, and in normal breast tissues (N). (**C**) *Correlation between TTK protein and RNA levels.* Omic data obtained in panels A and B reveal the consistency of TTK measurements between RNA and protein levels as quantified by Spearman correlation within the entire tumor population, within TNBC (**O**, red filled), Her2 (**Δ**), LB (**X**) and LA (**+**) tumors. (**D**) *Quantitative analysis of the percentage of TTK positive cells measured by immunohistochemistry (IHC) in human breast cancer tissues.* We present the results as the percentage of cells positively stained for TTK within fractions of samples in each BC sub-types, with TNBC in red, Her2 in purple, LB in green and LA in blue. (**E**) *Cellular detection of TTK within breast cancer biopsies by IHC.* We give a representative example of TTK staining on tissue microarrays (TMA) for the different breast tumor subtypes (TNBC, Her2, LB and LA) and for healthy breast tissues (N). Moreover, we indicate for each the percentage of TTK-positive cells. For all images, the scale bar is 20 µm (**F**) *Correlation between TTK and Ki67 RNA levels within the TNBC subtype.* (**G**) *Genomic alterations at TTK locus in human breast cancers.* Box-plots illustrate gain or loss of *TTK* DNA copy number from our breast cancer cohort with TNBC, Her2, LA and LB tumors. (**H**) *Correlation between the levels of TTK transcript and TTK DNA copy number.* Omic data obtained in panels A and G reveal the consistency of TTK measurements between RNA and DNA copy number, with TNBC (**O**, red filled), Her2 (**Δ**), LB (**X**) and LA (**+**) tumors.

Transcriptomic analyses pointed out a significant higher expression of TTK, a protein kinase involved in the mitotic spindle checkpoint, in TNBC samples (mean(log2) = 9.1) compared to Her2 (mean(log2) = 7.5; p = 4.3×10^−7^), LB (mean(log2) = 6.0; p<2.2×10^−16^) and LA (mean(log2) = 3.2; p<2.2×10^−16^), or to normal tissue samples (mean(log2) = 2.8; p<2.2×10^−16^) (T test, Figure 1A). These data are in line with previous studies showing elevated TTK mRNA levels in human BC samples [19], particularly in TNBC biopsies [20], [21]. Using the RPPA technology to measure TTK protein levels, we found that TNBC display higher levels of TTK protein compared to Her2 (p = 4.4×10^−4^), LB (p = 3.3×10^−9^) and LA (p = 2.2×10^−8^) tumors (T test, Figure 1B). When we compared transcriptomic and proteomic data, we observed that TTK mRNA and protein levels were well correlated within the entire population (Spearman correlation = 0.62, p<2.2×10^−16^) and also within the TNBC subgroup (Spearman correlation = 0.70, p = 3.7×10^−6^) (Figure 1C). In agreement with RPPA data, those obtained from IHC analysis highlighted high TTK protein expression in TNBC tissues (Figure 1D). We observed a higher percentage of patient samples with positive cells for TTK staining in the TNBC group as compared to the others, Her2 (p = 3.5×10^−5^), LB (p = 4.0×10^−8^) and LA (p = 1. 7×10^−9^) (Wilcoxon test, Figure 1D). In addition, the TTK staining was stronger in TNBC tissues compared to Her2 (p = 1.0×10^−4^), LB (p = 1.7×10^−8^) and LA (p = 1.1×10^−9^) (Fisher test, data not shown). Some tumor cells showed a cytoplasmic and nuclear staining for TTK (Figure 1E), consistent with the fact that TTK expression is highly regulated during cell cycle progression, with a peak in G_2_/M phase [40]. Accordingly, we observed that TTK protein levels correlated positively with the proliferation marker Ki67 mRNA within the TNBC subgroup (Spearman correlation = 0.45, p = 3.5×10^−3^) (Figure 1F). We did not detect TTK in healthy breast tissues (Figure 1E). We then examined whether variations in TTK protein expression could arise from genomic alterations in our TNBC subpopulation. By performing genomic analyses using SNP arrays, we observed that the locus of *TTK* is frequently gained in TNBC, in comparison with Her2 (p = 0.10), LB (p = 3.4×10^−2^) and LA (p = 2.1×10^−6^) tumors (Fisher test; Figure 1G). We found that TTK mRNA levels are correlated with *TTK* DNA copy-number within the entire population (Spearman correlation = 0.65, p<2.2×10^−16^) and also within the TNBC subgroup (Spearman correlation = 0.57, p = 1.5×10^−4^) (Figure 1H). Together, these omic analyses point out that TTK is highly expressed in TNBC which is at least partially due to genomic gain.

### Low Expression of TTK is Associated with Poor Clinical Outcome within the Triple-negative Breast Cancer

**Figure 2:**
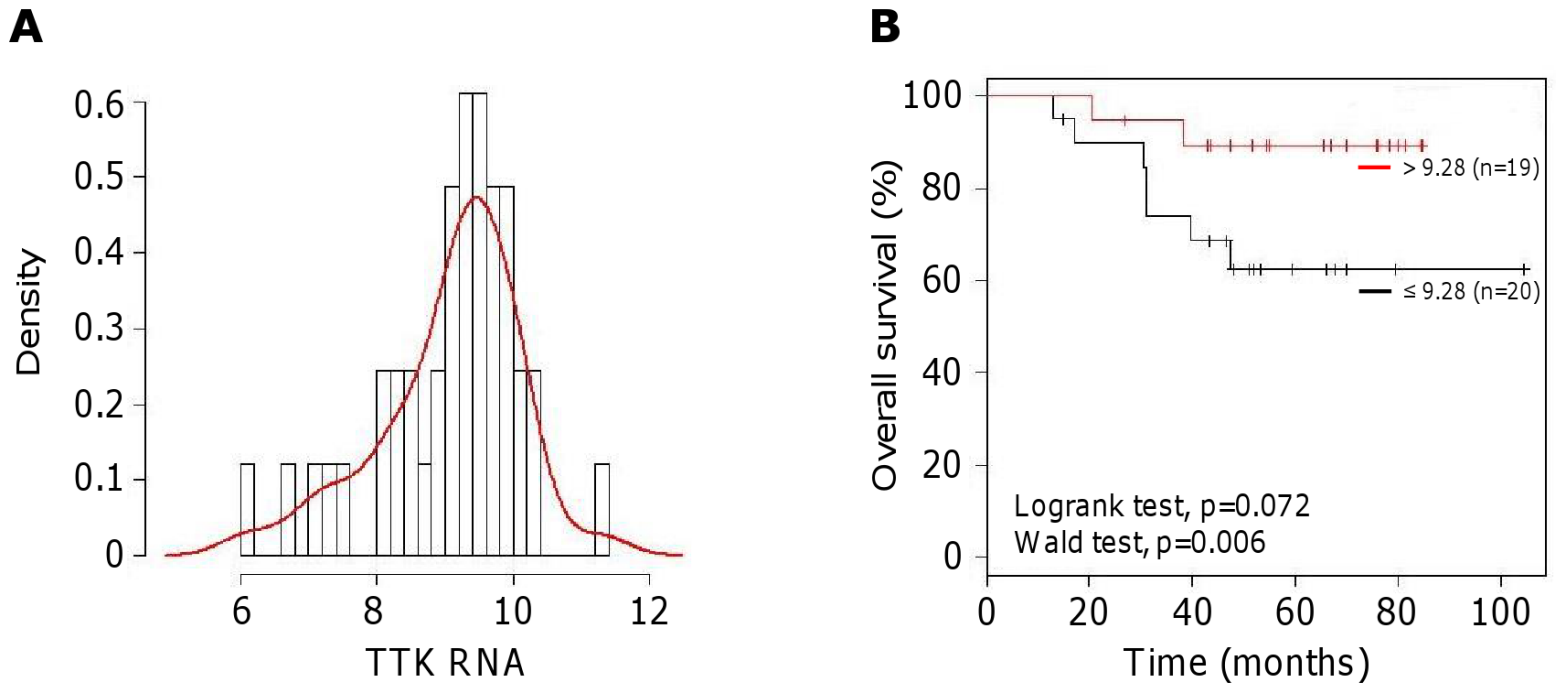
Correlation between TTK mRNA levels and overall survival in human triple-negative breast cancers. (**A**) *Distribution of TTK mRNA levels in TNBC patients*. We present the distribution of the logarithmic (log2) transformed levels of TTK RNA expression within the TNBC subgroup. (**B**) *Univariate Kaplan–Meier curves* of the overall survival in patients with TNBC divided into two groups, according to TTK mRNA expression dichotomized at the median value (≤9.28 and >9.28) to illustrate our data using the Wald test. We indicate the *p* values from Kaplan–Meier curves (hazard ratio = 3.33; 95% confidence interval = 0.9−12.39; p = 0.072, logrank test) and from the Cox regression model (p = 0.006).

By examining the clinical annotations related to our cohort, we addressed whether TTK expression levels were associated with disease outcome, as determined by the overall survival, the disease free interval (DFI) and the occurrence of metastasis. We found that high TTK expression is associated with poor prognosis within the entire population. However this simply reflected a higher expression of TTK in the poor prognosis-associated Her2 and TNBC subtypes. Therefore, we investigated the correlation between TTK expression and prognosis only within the TNBC population. No bimodal distribution of TTK expression was observed within the TNBC subgroup (Figure 2A) and we used a continuous variable test for the analysis. We found that low TTK expression in TNBC group is significantly associated in Cox proportional-hazard regression model with a poor overall survival, a higher metastasis propensity and a shorter DFI (p = 0.006, p = 0.013 and p = 0.045 respectively, Wald test). In order to illustrate these results, a logrank test was performed with TNBC patients divided into 2 groups based on their TTK expression levels, below and above the median (Figure 2B).

### siRNA-mediated Depletion of TTK in Triple-negative Breast Cancer Cell Lines Impairs Viability and Tumorigenicity

**Figure 3:**
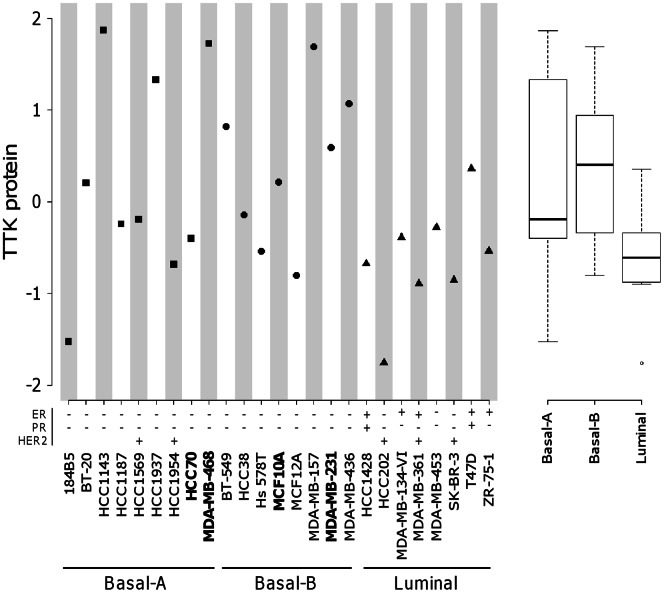
Expression of TTK protein in human cell lines derived from breast tissues. In left part, we show logarithmic (log2) transformed levels of TTK protein expression in our breast cell line collection. Breast cell lines are classified as basal-A, basal-B and luminal as in [41]. In addition, we present the ER/PR expression and the Her2 overexpression status as in [41]. We indicate in bold the cell lines used in our study. In the right part, the box-plots illustrate the logarithmic (log2) transformed levels of TTK protein within the breast cell line subgroups.

Given the fact that TTK plays a role in mitotic checkpoint by preventing chromosome instability and mitotic catastrophe, we investigated whether TTK depletion could selectively affect cell viability and tumorigenicity of triple negative breast cell lines. We first verified, with RPPA analysis, that TTK is expressed in a large panel of established cell lines derived from human breast tissues (Figure 3). We confirmed that TTK is highly expressed in basal-like cell lines compared to luminal cell lines (Figure 3). It is noteworthy that cells identified as basal-A or basal-B (Figure 3), following the classification from Neve and colleagues [41], contain TNBC cells, but also Her2 cell lines (HCC1569 and HCC1954) and non-tumorigenic cells (184B5, MCF10A and MCF12A). For *in vitro* analysis, we selected three TNBC cell lines, MDA-MB-468, MDA-MB-231 and HCC70 with high, intermediate and low TTK protein levels, respectively (Figure 3). These three cell lines are mutated for *TP53* gene (www.sanger.ac.uk/genetics/CGP/cosmic). In addition, we included in our study the non-malignant cell line, MCF10A, which is wild-type for *TP53*. We performed the depletion of TTK with 2 distinct and specific siRNAs (TTK_6 or TTK_7) and examined their effects on cell viability. The depletion of TTK was verified at the RNA level by quantitative-PCR (data not shown) and at the protein level by immunobloting.

**Figure 4:**
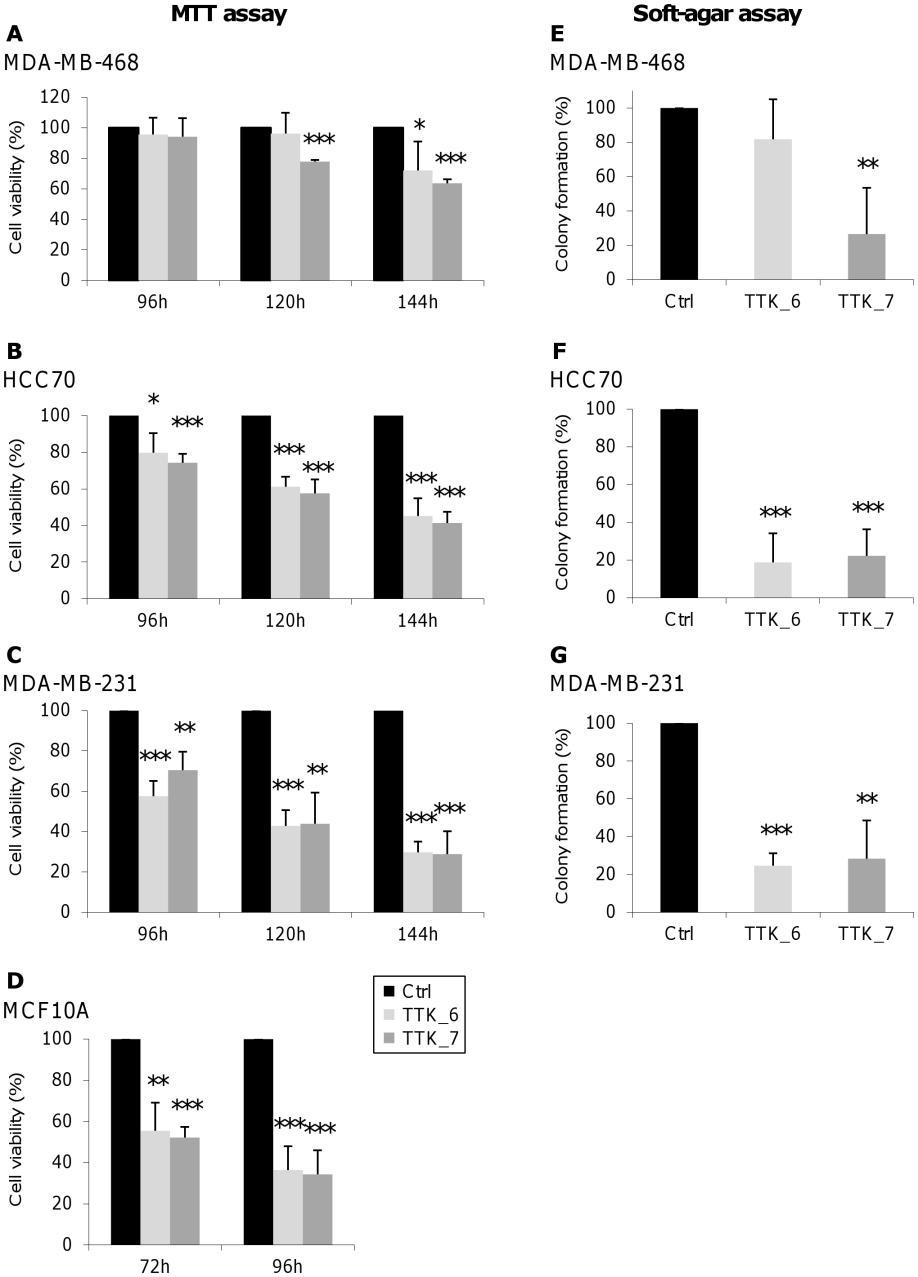
Viability of triple-negative breast cancer cell lines is impaired upon TTK depletion. (**A to D**) *Cell growth arrest after depletion of TTK.* We transfected established TNBC cell lines, MDA-MB-468 (A), HCC70 (B) and MDA-MB-231 (C), and non-malignant MCF10A cells (D) with control siRNA (Ctrl; black bars) or two siRNAs against TTK (TTK_6, grey bars and TTK_7; dark grey bars). We analyzed their capacity to grow at the indicated time points by MTT assay. Each value in the histograms corresponds to the percentage of cell viabilty relative to the control and represents the mean of at least three independent experiments. (**E to G**) *Impairment of clonogenicity after depletion of TTK.* We transfected TNBC cells, MDA-MB-468 (E), HCC70 (F) and MDA-MB-231 (G) with the indicated siRNAs, and analyzed their capacity to form colony in soft-agar (same representations as in panels A-D). The error bars represent the standard deviation of the mean and asterisks, the p values from Student’s *t* test (*p<0.05; **p<0.01; ***p<0.001).

We observed that the depletion of TTK significantly impaired the proliferation of the 3 TNBC cell lines, MDA-MB-468 (Figure 4A), HCC70 (Figure 4B) and MDA-MB-231 (Figure 4C). MDA-MB-468 cells appeared to be less affected by TTK depletion, especially with the TTK_6 siRNA, possibly due to the fact that these cells express very high levels of TTK (Figure 3) and that the depletion may not be efficient enough to observe more drastic effects. Our results are consistent with previous reports showing that TTK depletion reduces cell viability in a large variety of cancer cell lines [20], [23], [42], [43]. In contrast to a previous report [20], we observed similar effects with the “normal” MCF10A cells (Figure 4D). However, these results are in agreement with an inactivation of the SAC inducing an early exit from mitosis, leading to aberrant chromosome segregation and consequently to cell death. The fact that MCF10A cells grow much faster than the other cell lines, with a higher number of mitosis at the analyzed time points (data not shown), can explain why TTK depletion impairs MCF10A viability at earlier time points compared to the TNBC cell lines (Figure 4, A–D).

We then evaluated the effect of TTK silencing on the tumorigenic properties of TNBC cell lines through the anchorage-independent colony formation assay in soft agar. The non-malignant MCF10A cells do not have the ability to form colonies in these culture conditions, in contrast to some TNBC cell lines, such as MDA-MB-468, HCC70 and MDA-MB-231. We found that TTK siRNA-treated TNBC cells exhibited a remarkable decrease in their ability to form colonies in semi-solid medium (Figure 4, E–G). Again, TTK_6 siRNA was less effective on MDA-MB-468 cells (Figure 4E). Altogether, our results indicate that TTK plays an essential role in the malignancy of TNBC cells.

### TTK Depletion in Triple-negative Breast Cancer Cell Lines Affects Cell Cycle Progression

**Figure 5:**
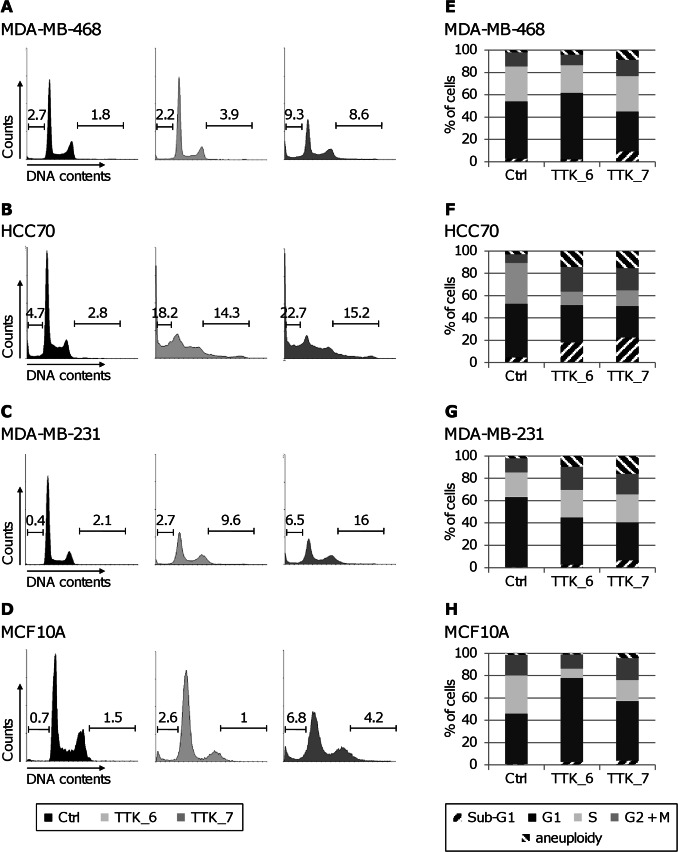
Depletion of TTK in triple-negative breast cancer cells affects cell cycle progression. (**A to D**) *Flow cytometry analysis of the cell cycle distribution after TTK depletion.* We transfected TNBC cells, MDA-MB-468 (A), HCC70 (B) and MDA-MB-231 (C), and normal MCF10A cells (D) with the indicated siRNAs, as in Figure 4. We monitored the cell cycle status of siRNA-treated cells by flow cytometry analysis following propidium iodide (PI) staining, 120 h for TNBC cells and 72 h for normal cells after siRNA-transfection. We also indicated for each profiles the percentage of sub-G1 cells on the left bars and the percentage of aneuploid cells on the right bars. (**E to H**) *Quantification of the cell cycle distribution after TTK depletion* corresponding to the panels A to D for MDA-MB-468 (E), HCC70 (F), MDA-MB-231 (G), and MCF10A (H) cells, with the percentages of cells in sub-G1 phase (right dark hatched), G1 phase (dark gray), S phase (light greys), G2+ M phase (grey) and aneuploid population (left black hatched).

Due to the role of TTK in the mitotic checkpoint, we investigated the consequences of TTK depletion on cell-cycle progression by FACS analysis. As the non-malignant MCF10A cells have much faster growth kinetics than all our TNBC cell lines, we analyzed the cell cycle profile at 72 h post-transfection for MCF10A cells and at 120 h post-transfection for TNBC cells. The transfection with control siRNA did not affect the cell cycle profile of TNBC and normal cells, compared to the corresponding untransfected cells (data not shown). In contrast, TTK-depleted cells showed an increase in the percentage of cells with aneuploid DNA content (Figure 5), in a more pronounced manner in the TNBC cells (Figure 5, A–C and E–G). This suggests that some cells depleted in TTK might exit mitosis earlier and thus undergo an abnormal cytokinesis, leading probably to aneuploidy. In addition, we observed an important decrease in the percentage of cells in G1 phase, a flattening in G2/M phase and, at the same time, a dramatic increase in the percentage of cells with sub-G1 DNA content (Figure 5). In contrast to TNBC, MCF10A cells depleted in TTK showed a substantial increase in G1 cell population, indicating a potential G1 arrest. Together, these results indicate that TTK depletion affects the progression through cell cycle and might induce mitotic catastrophe leading to cell death. Even if we harvested cells at different post-transfection times, the effects were more pronounced in TNBC cells (Figure 5, A–C and E–G) than in normal MCF10A cells (Figure 5, D and H). Indeed, the cell cycle profiles of MCF10A cells at 72 h, which grow twice faster than TNBC cells, with higher mitosis passages, seem to be less affected by TTK depletion than those of HCC70 and MDA-MB-231 cells at 120 h. In addition, it seems that TTK depletion rather induces an arrest in G1 phase in the non-tumorigenic MCF10A cells.

### TTK Depletion in Triple-negative Breast Cancer Cell Lines Induces Apoptosis

**Figure 6:**
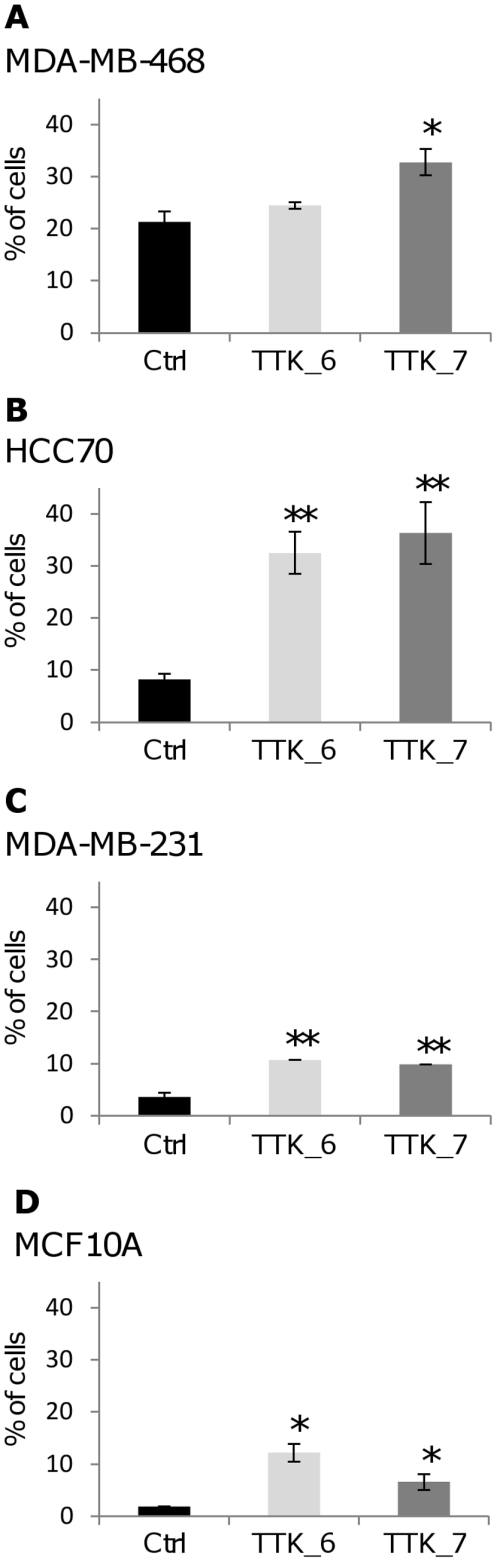
Induction of apoptosis by depletion of TTK in triple-negative breast cancer cells. We transfected MDA-MB-468 (**A**), HCC70 (**B**) and MDA-MB-231 (**C**) and MCF10A (**D**) cells with the indicated siRNAs. We analyzed the proportion of living and dead cells by FACs following PI and annexin V staining. Histograms with the percentage of apoptotic cells are presented, and are the mean of two to three independent experiments. We show the standard deviation of the mean by error bars and the p values from Student’s *t* test by asterisks (*p<0.05; **p<0.01).

**Figure 7:**
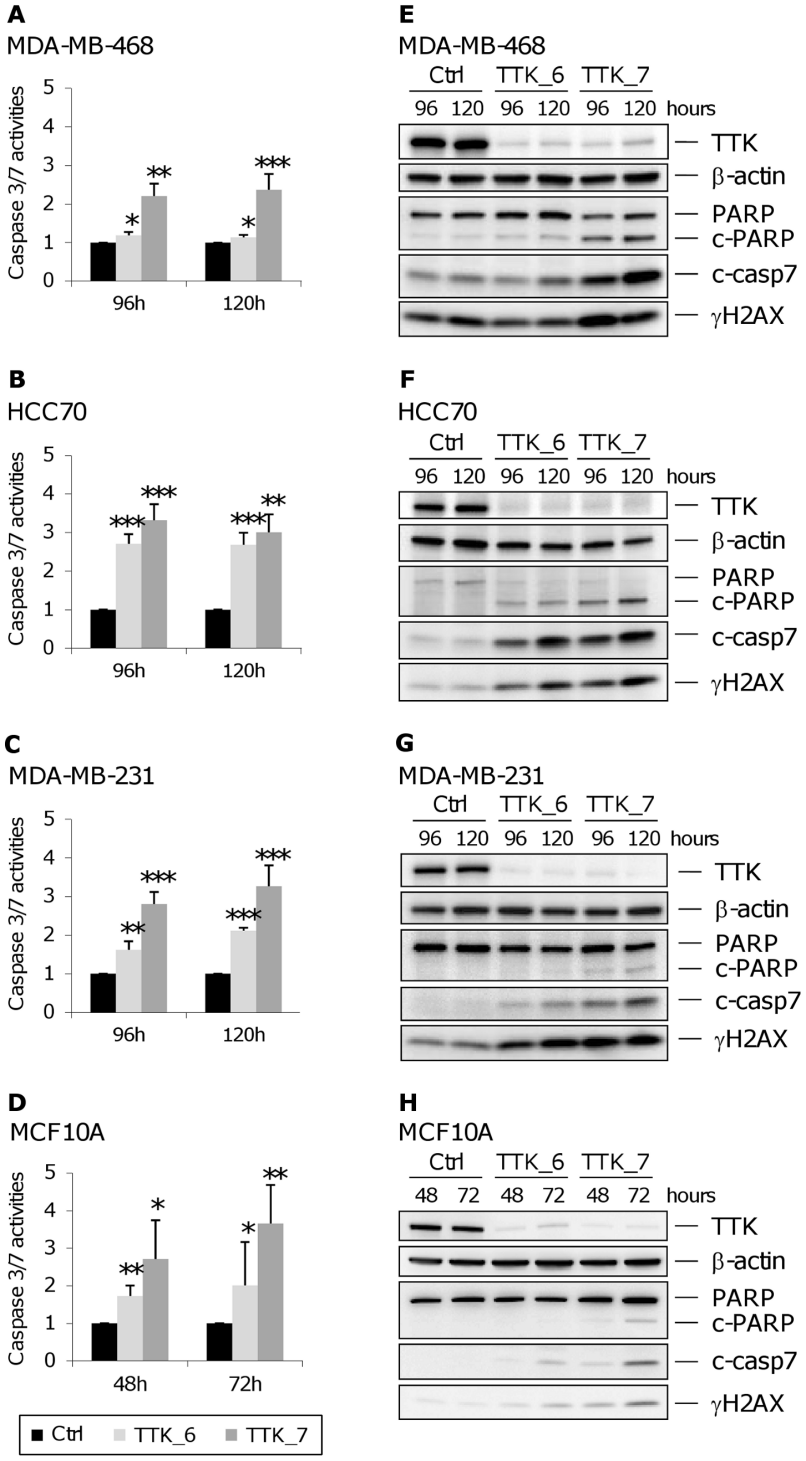
Activation of caspase 3/7 and cleavage of PARP following depletion of TTK in triple-negative breast cancer cells. (**A to D**) *Increased caspase 3/7 activity in TTK-depleted cells.* At the indicated time we measured caspase 3/7 activity in TNBC cells, MDA-MB-468 (A), HCC70 (B) and MDA-MB-231 (C), and normal MCF10A cells (D) following siRNA-transfection performed as in Figure 4. Each value in the histograms corresponds to the fold change in caspase 3/7 activity and represents the mean of three independent experiments. The error bars represent the standard deviation of the mean and the asterisks, the p values from Student’s *t* test (*p<0.05; **p<0.01; ***p<0.001). (**E to H**) *Increased cleavage of caspase 7 and PARP in TTK-depleted cells.* We transfected MDA-MB-468 (E), HCC70 (F), MDA-MB-231 (G) and MCF10A (H) cells with the indicated siRNAs. At the indicated time points, we harvested the cells and performed immunoblotting with antibodies against (i) cleaved PARP, which recognizes both uncleaved (PARP) and cleaved (c-PARP) forms, (ii) cleaved caspase 7 (c-casp7) and (iii) phosphorylated H2AX (γH2AX). We used an anti-TTK antibody to confirm TTK depletion and β-actin as a loading control.

We performed additional experiments to assay whether TTK-depleted cells undergo cell death by apoptosis. For the same reasons as previously mentioned, we measured the induction of apoptosis at different times, 72 h post-transfection for MCF10A cells and 120 h post-transfection for TNBC cells. First, we examined one of the earliest events in apoptosis, the externalization of phosphatidylserine by measuring annexin V staining in living cells. We found that TTK depletion increases the percentage of apoptotic cells both in normal and TNBC cells (Figure 6) whereas the percentage of necrotic cells did not change in comparison to cells transfected with the control siRNA (data not shown). We observed a more pronounced effect in HCC70 cells (Figure 6B), which is consistent with the FACs analysis (Figure 5B). These results strongly suggest that TTK silencing induces cell death by apoptosis. To further confirm this observation, we assessed the activity of effector caspases (Figure 7). Following TTK depletion, we observed an increase in caspase 3/7 activity (Figure 7, A–D). As the measurement of caspase 3/7 activity is linked to the number of cells that were present in the culture dish, we also analyzed caspase activity through the detection of the cleaved forms of caspase 7 on immunoblots after loading the same amount of protein lysates (Figure 7, E–H). As observed in the previous experiments, TTK_6 siRNA was less effective on MDA-MB-468 cells (Figure 7, A and E). To strengthen these data, we examined the cleaved form of PARP, a marker associated with apoptosis and cleaved by caspases during the execution phase of apoptosis. The levels of cleaved PARP were higher in TNBC cell lines compared to MCF10A (Figure 7, E–H), taken into account the initial amount of PARP detected in ctrl siRNA transfected cells. We also noticed a concomitant increase in the phosphorylation of histone variant H2AX (γH2AX). This histone modification is indicative of either formation of DNA breaks [44] during replication stress or abnormal mitotic chromosome segregation, or initiation of DNA fragmentation during apoptosis [45]. Increased γH2AX and cleaved forms of caspase 7, induced by TTK depletion, were more pronounced in TNBC cell lines than in MCF10A cells (Figure 7, E–H). Altogether, these data show that the depletion of TTK in TNBC cells leads to a strong reduction in cell viability as a result of an induction of apoptosis.

## Discussion

Among the BC subgroups, TNBC represents the major clinical challenge for oncologists [9]. Due to the absence of targeted therapies, patients with TNBC are treated exclusively with conventional cytotoxic therapies. While these tumors respond well to conventional chemotherapies, they relapse more frequently than the others BC subgroups. They are thus responsible for a disproportionate number of BC deaths, although TNBC accounts for about 15% of BC cases [10], [14], [15]. Therefore, the identification of relevant therapeutic targets in TNBC remains a critical issue in order to improve survival of such patients.

In order to discover new tailored therapeutic strategies in TNBC, we focused on protein kinases selectively overexpressed in human TNBC biopsies. From transcriptomic data obtained on human BC biopsies, we found that RNA levels of TTK were highly increased in TNBC samples compared to the other BC subgroups and healthy tissue samples. Our results are in agreement with previous microarray analyses showing that TTK is up-regulated in a variety of tumors, such as breast, bladder, esophagus, lung, prostate and anaplastic thyroid [19], [21], [46]–[49]. Moreover, TTK belongs to a signature defined by the top 25 genes overexpressed in chromosomally instable (CIN) and aneuploid tumors [50], highlighting their “addiction” to the mitotic checkpoint to sustain cell proliferation in the presence of CIN and aneuploidy. In addition, TTK belongs to a list of 16 kinases overexpressed in TNBC in comparison to LA breast cancers [21]. Recently, it has been shown that TTK mRNA expression correlates with high histologic grade in BC, and that high TTK mRNA levels are found in TNBC [19], [20]. Here, we report for the first time that TTK is overexpressed at the protein level in TNBC compared to the other BC subgroups, by IHC and RPPA techniques. We found that high TTK expression is associated with poor prognosis within the entire population. However this simply reflected the high expression of TTK in the poor prognosis-associated Her2 and TNBC subtypes. Within our TNBC subpopulation, higher TTK expression is associated with a better prognosis. This may be explained by the fact that low TTK expressing tumors are less responsive to conventional chemotherapy, possibly due to their lowest proliferative rates, as we show a positive correlation between TTK and the proliferation marker Ki67. This is in agreement with our recent observation that higher expression of Polo-like kinase 1 (PLK1), another protein kinase whose expression is correlated to proliferation, is associated with better prognosis within the TNBC subgroup [51]. Moreover, we found that low Ki67 mRNA is correlated with poorer prognosis within the TNBC subgroup (data not sown).

Since others have suggested that inhibition of mitotic checkpoint could have therapeutic potential in cancer treatment [25], [26], we decided to focus on the potential deleterious effect of TTK silencing on TNBC cell lines. In agreement with others studies performed on tumor cell lines of various origins [20], [23], [52], we found that the RNAi-mediated depletion of TTK severely compromises the viability of human TNBC cells. However, in contrast to Daniel et al. [20], we also found that TTK depletion strongly affects the viability of non-tumorigenic MCF10A cells, as shown by Kwiatkowski et al. when they used small-molecule kinase inhibitors [42]. Our results are in agreement with the function of TTK in SAC, which enables cells to delay cell cycle progression from metaphase to anaphase before attachment of every chromosome to spindle microtubules [22], preventing chromosome mis-segregation, aneuploidy and cell death [23], [24]. Our data suggest that depletion of TTK would selectively impair growth of rapidly dividing cells, one of the hallmarks of tumor cells. In addition, we showed that TTK depletion in TNBC cells decreases their capacity to form colonies in an anchorage-independent manner, another hallmark of malignant cells. Moreover, TTK depletion leads to cell death by apoptosis, as shown by an elevation of annexin-V positive cells and a sub-G1 associated-cell population, as well as an increased of caspase 3/7 activities and PARP cleavage.

Our results are in agreement with previous data showing that absence of other mitotic regulators, such as PLK1 or Aurora kinase family members, affects preferentially cancer cell proliferation [53], [54]. Thus, the differences that we found between normal and tumor cells in cell cycle progression and induction of apoptosis upon TTK depletion might reflect differences in genomic background. One explanation may involve the p53 protein, which is also implicated among others in the post-mitotic checkpoint to prevent aneuploidy [55], [56]. Recently, it has been suggested that phosphorylation of p53 at threonine 18 (T18) by TTK may contribute to the post-mitotic checkpoint, which arrests cells in G1 phase and therefore prevents further polyploidization [57]. Similarly to TNBC tumors, in which *TP53* gene is frequently mutated [58], the three TNBC cell lines used in this study are also mutated for *TP53* (www.sanger.ac.uk/genetics/CGP/cosmic). This may explain why we observed, following TTK depletion, a higher percentage of aneuploid cells in TNBC cell lines compared to normal cells that seem to arrest in G1, in agreement with a previous study [57].

In addition, TTK depletion in TNBC cell lines leads to an increase in H2AX phosphorylation. γH2AX is generally used to monitor the presence of DNA double strand breaks (DSBs) within the nucleus. Upon TTK depletion, increase of γH2AX could reflect either DNA fragmentation at the time of apoptosis initiation [45], or chromosome breaks that can occur during aberrant mitosis [59]. It has been shown recently that TTK plays a role in DNA damage response. TTK binds and phosphorylates CHK2 kinase [60], enhancing the activation of DNA damage G2/M checkpoint. Moreover, in mitosis, TTK promotes phosphorylation of Bloom syndrome protein (BLM) and subsequent interaction of BLM with PLK1, ensuring accurate chromosome segregation [61]. Thus, DNA breaks induced during aberrant mitosis due to the absence of TTK would be inefficiently repaired in the presence of a defective cell cycle arrest mediated by CHK2, BLM, PLK1 or p53. TNBC is associated with a “BRCAness” phenotype, meaning that DSB repair through homologous recombination (HR), one of the two major DSB repair mechanism, is deficient [62]–[64]. Moreover, TNBC are also deficient for PTEN [29], [65]–[67], which was recently implicated in the regulation of HR [68]. Some TTK-depleted TNBC cells seem to go through mitosis, as shown by the increased proportion of aneuploid cells. However, with weakened checkpoints and inefficient HR-mediated DSB repair, these cells cannot maintain prolonged cell cycle blockage and enter in next mitosis before mitotic DNA lesions can be repaired, leading to a massive mitotic apoptosis. This may explain the high increase of sub-G1 cells and the important decrease of G1 cells in TNBC cells, compared to MCF10A, following TTK depletion. In other terms, the absence of TTK in TNBC cells might exacerbate their genomic instability, eventually leading to death. Our results suggest that the effects of TTK depletion on cell proliferation and viability are probably not only due to its role in mitotic checkpoint but also to its involvement in DNA damage response. Consistently, TTK has been recently found to form a synthetic lethal pair with PTEN [65], suggesting that TTK could represent an attractive target in PTEN-deficient associated TNBC, providing a therapeutic window for targeting TTK in TNBC patients without affecting normal proliferative tissues. However, from its reported mRNA expression, we cannot exclude possible side effects in proliferative tissues, such as bone marrow or stomach (see supplementary table 3 from [69]).

Altogether, our data suggest that the targeting of TTK may represent a promising approach for patients with TNBC. However, as most of the studies so far have used nonselective inhibitors or inhibitors with different effects on the localization of SAC proteins to kinetochore [26], [52], [70], it would be of interest to confirm our *in vitro* results using specific TTK inhibitors *in vivo* in human TNBC-derived xenograft models [71].
